# Data and calculus on isobolographic analysis to determine the antinociceptive interaction between calcium channel blocker and a TRPV1 blocker in acute pain model in mice

**DOI:** 10.1016/j.dib.2017.07.059

**Published:** 2017-07-27

**Authors:** Juliana F. Silva, Manuella R. Palhares, Duana C. Santos, Cláudio A. Silva-Junior, Juliano Ferreira, Marcus V. Gomez, Célio J. Castro Junior

**Affiliations:** a Department of Neurotransmitters, Institute of Education and Research, Santa Casa, Belo Horizonte, Minas Gerais 30150-240, Brazil; b Department of Pharmacology, Biological Sciences Center, Federal University of Santa Catarina, Florianópolis, Santa Catarina 80040-900, Brazil

## Abstract

Determining antinociceptive interaction between Phα1β toxin (a voltage gated calcium channel blocker) and SB366791 (selective TRPV1 antagonist) may have both clinical and mechanistic implications for the pain management. This data in brief article is associated to the research paper “Synergistic antinociceptive effect of a calcium channel blocker and a TRPV1 blocker in an acute pain model in mice”. This material supports the isobolographic analysis performed with the above drugs and shows: data of the dose response curves of the agents given as single drug or combination regimens. Mathematics and statistical processing of dose response curves, proportion of drugs dosage to be used in the combination, calculus of theoretical additive DE_20_ dose as well as experimentally obtained DE_20_ are provided. It is also presented details of statistical comparison between theoretical and experimentally obtained DE_20_.

**Specifications Table**

**Table:** 

Subject area	*Biology*
More specific subject area	*Neuropharmacology, pain management*
Type of data	*Table, text file*
How data was acquired	*Experimental observation of nociceptive behavior in mice by using a chronometer.*
Data format	*Raw and analyzed*
Experimental factors	*Dose response curves for Single drug administration of combined drugs, given at the same of at different sites*
Experimental features	*Isobolographic analysis using a fixed proportion ratio of two drugs.*
Data source location	*N.A.*
Data accessibility	*Data is with this article.*

**Value of the data**

– *Data tables and calculus from this article may serves as a practical guide on isobolographic analysis for testing other drug regimens of even using different pain models.*
– *Determining the interaction index by isobolographic analysis provides a measure of the in vivo degree of interaction of two drugs for a specified effect.*
– *Data analysis presented in this article can be systematically compared to other data from probit analysis with analgesic drugs given in combination.*

## 1 Data

### 1.1 Summary of linear regression analyses for the agents administered alone

#### 1.1.1 Intraplantar SB366791

See Table 1.

**Table 1:** Dose-effect data of SB366791 (i.p.) on capsaicin-induced nociception.

Dose	x_i_	y_i_	x_i_.y_i_	(x_i_-$\overset{®}{X}$)^2^
0.10	-1.00	8.86	-8.86	0.74
0.10	-1.00	-14.77	14.77	0.74
0.10	-1.00	-41.77	41.77	0.74
0.10	-1.00	38.29	-38.29	0.74
0.10	-1.00	37.43	-37.43	0.74
0.10	-1.00	-22.57	22.57	0.74
0.10	-1.00	-23.58	23.58	0.74
0.10	-1.00	0.81	-0.81	0.74
0.10	-1.00	-10.76	10.76	0.74
0.10	-1.00	-0.63	0.63	0.74
0.10	-1.00	-13.83	13.83	0.74
0.10	1.00	64.31	-64.31	0.74
0.10	-1.00	48.42	-48.42	0.74
0.10	-1.00	18.18	-18.18	0.74
0.40	-0.40	-13.08	5.23	0.07
0.40	-0.40	-23.21	9.28	0.07
0.40	-0.40	4.64	-1.86	0.07
0.40	-0.40	9.14	-3.66	0.07
0.40	-0.40	36.57	-14.63	0.07
0.40	-0.40	27.14	-10.86	0.07
0.40	-0.40	45.80	-18.32	0.07
0.40	-0.40	33.33	-13.33	0.07
0.40	-0.40	0.27	-0.11	0.07
0.40	-0.40	12.03	-4.81	0.07
0.40	-0.40	59.49	-23.80	0.07
1.00	0.00	-25.75	0.00	0.02
1.00	0.00	18.16	0.00	0.02
1.00	0.00	12.20	0.00	0.02
1.00	0.00	-1.90	0.00	0.02
1.00	0.00	17.72	0.00	0.02
1.00	0.00	25.32	0.00	0.02
1.00	0.00	2.53	0.00	0.02
1.00	0.00	5.06	0.00	0.02
1.00	0.00	-19.61	0.00	0.02
1.00	0.00	6.43	0.00	0.02
1.00	0.00	-12.06	0.00	0.02
1.00	0.00	50.79	0.00	0.02
1.00	0.00	29.37	0.00	0.02
1.00	0.00	15.08	0.00	0.02
1.00	0.00	44.44	0.00	0.02
1.00	0.00	31.75	0.00	0.02
2.00	0.30	26.58	7.97	0.19
2.00	0.30	70.46	21.14	0.19
2.00	0.30	53.59	16.08	0.19
2.00	0.30	22.86	6.86	0.19
2.00	0.30	40.86	12.26	0.19
2.00	0.30	13.43	4.03	0.19
2.00	0.30	13.28	3.98	0.19
2.00	0.30	22.49	6.75	0.19
2.00	0.30	52.85	15.85	0.19
2.00	0.30	-10.13	-3.04	0.19
6.00	0.78	30.62	23.89	0.85
6.00	0.78	65.31	50.94	0.85
6.00	0.78	17.07	13.32	0.85
6.00	0.78	84.28	65.74	0.85
6.00	0.78	25.95	20.24	0.85
6.00	0.78	23.42	18.27	0.85
6.00	0.78	18.99	14.81	0.85
6.00	0.78	6.33	4.94	0.85
6.00	0.78	60.13	46.90	0.85

**Legend:** Dose (nmol/site); x_i_: Log of dose (nmol/site); y_i_: (% of MEP); x_i_y_i_: product x_i_.y_i_; (x_i_-$\overset{®}{X}$)^2^: (x_i_ – x_i_ average)^2^.

-Linear regression summary:

∑x= -8.38; ∑y= 1118.42; N= 60; $\overset{®}{X}$= -0.1397; $\overset{®}{Y}$=18.64; ∑x^2^ = 22.13;

∑x.y= 185.67; N.x.y = -156.20; $\overset{®}{X}$^2^= 0.0195

b= $\frac{\sum \mathrm{xiyi}-N\overset{®}{\mathrm{xy}}}{\Sigma {\mathrm{xi}}^{2}-N{\overset{®}{x}}^{2}}$;

*a* = $\overset{®}{y}$ - b$\overset{®}{x}$. thus *b* = 16.3 (slope) and *a* = 20.9 *(intercept)*

-Slope variance = $V(b)=\frac{s^{2}}{Ƹ\left(\mathrm{xi}-X\right)2}$. wherein s^2^ = *Q/(N-2*) and *Q* = $\sum_{i=1}^{N}(yi–a–\mathrm{bx}i)$

V(b) = 31.3

- Assessment if slope differs from 0:

t_*slope*_ = b/{V(b)}^1/2^; t_*slope*_ = 2.91 which exceeds t-tabulated, thus slope differs from zero.

t_*table*_ (N-2 degrees of freedom; P=0.05; from t-distribution) = 1.96

- DE_20_ calculation: DE20 = (20 – a)/b (in log scale); DE20 = −0.056 or 0.88 nmol/site

- Variance of DE_20_ (V_DE20_); = s^2^ / b^2^ [ 1/N + (x^1^−x¨)^2^/ S_xx_]; V_DE20_ = 0.042 (in log scale)

#### 1.1.2 Intraplantar Phα1β

See Table 2.

**Table 2:** Dose-effect data Phα1β (i.p.) on capsaicin-induced nociception.

Dose	x_i_	y_i_	x_i_y_i_	(x_i_−X̅)2
0.01	-2.00	19.51	-39.02	1.00
0.01	-2.00	27.44	-54.88	1.00
0.01	-2.00	46.34	-92.68	1.00
0.01	-2.00	0.33	-0.66	1.00
0.01	-2.00	6.23	-12.46	1.00
0.01	-2.00	-36.30	72.61	1.00
0.01	-2.00	20.49	-40.98	1.00
0.01	-2.00	12.31	-24.63	1.00
0.03	-1.52	18.90	-28.73	0.27
0.03	-1.52	31.71	-48.20	0.27
0.03	-1.52	18.69	-28.41	0.27
0.03	-1.52	44.92	-68.28	0.27
0.03	-1.52	7.54	-11.46	0.27
0.03	-1.52	30.51	-46.38	0.27
0.03	-1.52	30.51	-46.38	0.27
0.03	-1.52	-15.59	23.70	0.27
0.10	-1.00	64.63	-64.63	0.00
0.10	-1.00	26.22	-26.22	0.00
0.10	-1.00	50.61	-50.61	0.00
0.10	-1.00	48.17	-48.17	0.00
0.10	-1.00	8.20	-8.20	0.00
0.10	-1.00	14.10	-14.10	0.00
0.10	-1.00	35.74	-35.74	0.00
0.10	-1.00	37.05	-37.05	0.00
0.10	-1.00	25.84	-25.84	0.00
0.10	-1.00	29.18	-29.18	0.00
0.10	-1.00	38.53	-38.53	0.00
0.30	-0.52	54.27	-28.22	0.23
0.30	-0.52	44.51	-23.15	0.23
0.30	-0.52	43.61	-22.68	0.23
0.30	-0.52	20.00	-10.40	0.23
0.30	-0.52	30.49	-15.86	0.23
0.30	-0.52	45.88	-23.86	0.23
0.30	-0.52	55.23	-28.72	0.23
0.30	-0.52	5.12	-2.66	0.23
0.50	-0.30	67.68	-20.30	0.49
0.50	-0.30	68.29	-20.49	0.49
0.50	-0.30	62.20	-18.66	0.49
0.50	-0.30	62.80	-18.84	0.49
0.50	-0.30	65.85	-19.76	0.49
0.50	-0.30	30.49	-9.15	0.49
0.50	-0.30	35.08	-10.52	0.49
0.50	-0.30	41.64	-12.49	0.49
0.50	-0.30	41.64	-12.49	0.49
0.50	-0.30	41.87	-12.56	0.49
0.50	-0.30	58.57	-17.57	0.49
0.50	-0.30	56.57	-16.97	0.49

**Legend:** Dose (nmol/site); x_i_: Log of dose (nmol/site); y_i_: (% of MEP); x_i_y_i_: product x_i_.y_i_; (x_i_−$\overset{®}{X}$)^2^: (x_i_ –x_i_ averaged)^2^.

Linear regression summary:

∑x= -46.92; ∑y= 1589.6; N= 47; $\overset{®}{X}$= -0.99; $\overset{®}{Y}$= 33.82; ∑x^2^ = 64.72;

∑x.y= -1175.24; N.x.y = -1586.9; $\overset{®}{X}$^2^= 0.99

b= $\frac{\sum \mathrm{xiyi}-N\overset{®}{\mathrm{xy}}}{\Sigma {\mathrm{xi}}^{2}-N{\overset{®}{x}}^{2}}$;

*a* = $\overset{®}{y}$ - b$\overset{®}{x}$. thus *b* = 23.^2^ (slope) and *a* = 57.1 *(intercept)*

Slope variance = $V(b)=\frac{s^{2}}{Ƹ\left(\mathrm{xi}-X\right)2}$. wherein s^2^ = *Q/(N-2*) and *Q* = $\sum_{i=1}^{N}(yi–a–\mathrm{bx}i)$

V(b) = 18.1

- Assessment if slope differs from 0:

t_*slope*_ = b/{V(b)}^1/2^; t_*slope*_ = 5.40 which exceeds t-tabulated, thus slope differs from zero

t_*table*_ (N-2 degrees of freedom; P=0.05; from t-distribution) = 1.96

- DE_20_ calculation: DE20 = (20 – a)/b (in log scale); DE20 = −1.60 or 0.025 nmol/site

- Variance of DE_20_ (V_DE20_); = s^2^ / b^2^ [ 1/N + (x^1^−x¨)^2^/ S_xx_]; V_DE20_ = 0.025 (in log scale)

#### 1.1.3 Intrathecal Phα1β

See Table 3.

**Table 3:** Dose-effect data of Phα1β (i.t.) on capsaicin-induced nociception.

Dose	x_i_	y_i_	x_i_y_i_	(x_i_- X̅)^2^
0.0003	-3.52	39.54	-139.20	2.47
0.0003	-3.52	-25.58	90.05	2.47
0.0003	-3.52	31.40	-110.50	2.47
0.0003	-3.52	-4.65	16.37	2.47
0.0003	-3.52	41.29	-145.30	2.47
0.0003	-3.52	13.11	-46.15	2.47
0.0003	-3.52	4.50	-15.84	2.47
0.0003	-3.52	-0.98	3.44	2.47
0.0003	-3.52	13.82	-48.64	2.47
0.0003	-3.52	12.88	-45.34	2.47
0.0003	-3.52	-1.17	4.12	2.47
0.0003	-3.52	20.38	-71.72	2.47
0.0003	-2.52	50.00	-126.00	0.33
0.003	-2.52	24.63	-62.06	0.33
0.003	-2.52	39.55	-99.67	0.33
0.003	-2.52	34.18	-86.13	0.33
0.003	-2.52	65.40	-164.80	0.33
0.003	-2.52	20.37	-51.33	0.33
0.003	-2.52	24.07	-60.67	0.33
0.003	-2.52	17.44	-43.95	0.33
0.003	-2.52	23.26	-58.60	0.33
0.003	-2.52	20.93	-52.74	0.33
0.003	-2.52	27.20	-68.55	0.33
0.003	-2.52	63.47	-159.90	0.33
0.003	-2.52	42.86	-108.00	0.33
0.003	-2.52	37.24	-93.84	0.33
0.01	-2.00	19.84	-39.68	0.00
0.01	-2.00	-30.95	61.91	0.00
0.01	-2.00	47.76	-95.52	0.00
0.01	-2.00	50.00	-100.00	0.00
0.01	-2.00	96.27	-192.50	0.00
0.01	-2.00	40.08	-80.17	0.00
0.01	-2.00	33.33	-66.67	0.00
0.01	-2.00	26.58	-53.16	0.00
0.01	-2.00	26.19	-52.38	0.00
0.01	-2.00	41.67	-83.33	0.00
0.01	-2.00	37.96	-75.93	0.00
0.01	-2.00	35.19	-70.37	0.00
0.01	-2.00	43.52	-87.04	0.00
0.01	-2.00	43.52	-87.04	0.00
0.01	-2.00	45.37	-90.74	0.00
0.01	-2.00	50.00	-100.00	0.00
0.10	-1.00	50.00	-50.00	0.90
0.10	-1.00	36.51	-36.51	0.90
0.10	-1.00	39.55	-39.55	0.90
0.10	-1.00	69.40	-69.40	0.90
0.10	-1.00	68.66	-68.66	0.90
0.10	-1.00	29.11	-29.11	0.90
0.10	-1.00	40.93	-40.93	0.90
0.10	-1.00	28.27	-28.27	0.90
0.10	-1.00	33.33	-33.33	0.90
0.10	-1.00	58.33	-58.33	0.90
0.10	-1.00	42.59	-42.59	0.90
0.10	-1.00	66.67	-66.67	0.90
0.10	-1.00	55.56	-55.56	0.90
0.10	-1.00	59.26	-59.26	0.90
0.10	-1.00	69.44	-69.44	0.90
0.30	-0.52	57.14	-29.71	2.04
0.30	-0.52	-3.97	2.06	2.04
0.30	-0.52	58.21	-30.27	2.04
0.30	-0.52	71.64	-37.25	2.04
0.30	-0.52	67.91	-35.31	2.04
0.30	-0.52	45.15	-23.48	2.04
0.30	-0.52	51.06	-26.55	2.04
0.30	-0.52	29.96	-15.58	2.04
0.30	-0.52	36.91	-19.19	2.04
0.30	-0.52	60.71	-31.57	2.04
0.30	-0.52	51.85	-26.96	2.04
0.30	-0.52	75.93	-39.48	2.04
0.30	-0.52	55.56	-28.89	2.04
0.30	-0.52	62.96	-32.74	2.04
0.30	-0.52	77.78	-40.44	2.04

Legend: Dose (nmol/site); x_i_: Log of dose (nmol/site); y_i_: (% of MEP); x_i_y_i_: product x_i_.y_i_; (x_i_−$\overset{®}{X}$)^2^: (x_i_ –x_i_ averaged)^2^.

- Linear regression summary:

∑x= -132.32; ∑y= 2787; N= 72; $\overset{®}{X}$= -1.84; $\overset{®}{Y}$= 38.72; ∑x^2^ = 320.64;

∑x.y= -4120; N.x.y = -5123; $\overset{®}{X}$^2^= 3.38

b= $\frac{\sum \mathrm{xiyi}-N\overset{®}{\mathrm{xy}}}{\Sigma {\mathrm{xi}}^{2}-N{\overset{®}{x}}^{2}}$;

*a* = $\overset{®}{y}$ - b$\overset{®}{x}$. thus *b* = 12.9 (slope) and *a* = 62.5 *(intercept)*

- Slope variance = $V(b)=\frac{s^{2}}{Ƹ\left(\mathrm{xi}-X\right)2}$. wherein s^2^ = *Q/(N-2*) and *Q* = $\sum_{i=1}^{N}(yi–a–\mathrm{bx}i)$

V(b) = 4.9

- Assessment if slope differs from 0:

t_*slope*_ = b/{V(b)}^1/2^; t_*slope*_ = 5.84 which exceeds t-tabulated, thus slope differs from zero.

t_*table*_ (N-2 degrees of freedom; P=0.05; from t-distribution) = 1.96

- DE_20_ calculation: DE20 = (20 – a)/b (in log scale); DE20 = −3.28 or 0.5 pmol/site

- Variance of DE_20_ (V_DE20_); = s^2^ / b^2^ [ 1/N + (x^1^−x¨)^2^/ S_xx_]; V_DE20_ = 0.09 (in log scale)

### 1.2 Calculation of the proportion of constituents in the mixture

a + b = c. wherein:
a = the quantity (in nmol) of SB366791
b = quantity (nmol) of Phα1β
c = sum (in nmol) of quantities of Phα1β and SB366791 in the mixture.

The proportion of a and b was fixed and calculated according to the formulae below:

a = A x f
b = (1-f) x B; wherein:
A = DE_20_ of SB366791
B = DE_20_ of Phα1β
f = proportion factor.

The factor f was calculated based on the variances of the DE_20_ values from SB366791 (A) and Phα1β (B) according to the formula:

f = V (B) / V (A) + V (B); wherein:
V (A) = variance of DE_20_ of SB366791 and
V (B) = variance of DE_20_ of Phα1β;

– For combination of intraplantar Phα1β with SB366791. f = 0.38
– For combination of intraplantar SB366791 with intrathecal Phα1β. f = 0.69 (Table 4, Table 5).

**Table 4:** Doses used in the dose-response curve for intraplantar combination of SB366791 and Phα1β.

Drug pair	SB366791 (nmol/site)	Phα1β (nmol/site)	Composed drug pair (nmol/site)
1	0.012	0.0006	0.013
2	0.037	0.001	0.038
3	0.11	0.005	0.115
4	0.33	0.016	0.346
5	0.99	0.048	1.038
6	2.97	0.144	3.114

**Table 5:** Doses used in the dose-response curve for concurrent treatment with SB366791 (i.p.) and Phα1β (i.t.).

Drug pair	SB366791 (nmol/site)	Phα1β (nmol/site)	Composed drug pair (nmol/site)
1	0.022	0.00000048	0.02200048
2	0.066	0.0000014	0.0660014
3	0.2	0.0000043	0.2000043
4	0.60	0.00013	0.60013
5	1.8	0.00039	1.80039
6	5.4	0.00117	5.40117

## 1.3 Summary of linear regression analyses for the agents administered in combination

### 1.3.1 Phα1β (i.p.) combined with SB366791 (i.p.)

 See Table 6.

**Table 6:** Dose-effect data of Phα1β (i.p.) combined with SB366791 (i.p.) on capsaicin-induced nociception.

Dose	x_i_	y_i_	x_i_y_i_	(x_i_−X̅)2
0.013	-1.92	34.00	-64.79	1.57
0.013	-1.92	24.85	-47.71	1.57
0.013	-1.92	3.93	-7.54	1.57
0.013	-1.92	27.50	-52.81	1.57
0.013	-1.92	-19.89	38.19	1.57
0.013	-1.92	-2.37	4.55	1.57
0.013	-1.92	32.66	-62.72	1.57
0.013	-1.92	-63.64	122.18	1.57
0.013	-1.92	25.00	-48.00	1.57
0.013	-1.92	29.09	-55.85	1.57
0.013	-1.92	-20.00	38.40	1.57
0.013	-1.92	25.68	-49.31	1.57
0.038	-1.42	36.81	-52.27	0.57
0.038	-1.42	43.56	-61.85	0.57
0.038	-1.42	26.38	-37.46	0.57
0.038	-1.42	30.86	-43.82	0.57
0.038	-1.42	29.27	-41.57	0.57
0.038	-1.42	44.01	-62.49	0.57
0.038	-1.42	26.09	-37.05	0.57
0.038	-1.42	39.23	-55.71	0.57
0.038	-1.42	38.14	-54.16	0.57
0.038	-1.42	27.05	-38.40	0.57
0.038	-1.42	20.23	-28.72	0.57
0.038	-1.42	16.82	-23.88	0.57
0.115	-0.93	15.95	-14.83	0.07
0.115	-0.93	46.01	-42.79	0.07
0.115	-0.93	27.61	-25.67	0.07
0.115	-0.93	50.10	-46.59	0.07
0.115	-0.93	44.09	-41.00	0.07
0.115	-0.93	28.09	-26.13	0.07
0.115	-0.93	32.22	-29.96	0.07
0.115	-0.93	46.37	-43.12	0.07
0.115	-0.93	26.09	-24.27	0.07
0.115	-0.93	41.42	-38.52	0.07
0.115	-0.93	37.59	-34.96	0.07
0.115	-0.93	31.82	-29.59	0.07
0.115	-0.93	25.00	-23.25	0.07
0.115	-0.93	16.00	-15.01	0.07
0.115	-0.93	16.82	-15.64	0.07
0.346	-0.46	51.53	-23.71	0.04
0.346	-0.46	52.15	-23.99	0.04
0.346	-0.46	31.90	-14.67	0.04
0.346	-0.46	54.31	-24.98	0.04
0.346	-0.46	51.30	-23.60	0.04
0.346	-0.46	59.12	-27.19	0.04
0.346	-0.46	40.01	-18.40	0.04
0.346	-0.46	33.99	-15.63	0.04
0.346	-0.46	46.90	-21.57	0.04
0.346	-0.46	60.04	-27.62	0.04
0.346	-0.46	74.27	-34.16	0.04
0.346	-0.46	35.91	-16.52	0.04
0.346	-0.46	30.45	-14.01	0.04
0.346	-0.46	36.59	-16.83	0.04
0.346	-0.46	15.45	-7.11	0.04
1.038	0.02	52.76	0.84	0.47
1.038	0.02	60.12	0.96	0.47
1.038	0.02	53.99	0.86	0.47
1.038	0.02	86.77	1.39	0.47
1.038	0.02	49.50	0.79	0.47
1.038	0.02	63.93	1.02	0.47
1.038	0.02	54.03	0.86	0.47
1.038	0.02	45.78	0.73	0.47
1.038	0.02	49.90	0.80	0.47
1.038	0.02	49.64	0.79	0.47
1.038	0.02	65.51	1.05	0.47
1.038	0.02	59.49	0.95	0.47
1.038	0.02	45.45	0.73	0.47
1.038	0.02	45.45	0.73	0.47
1.038	0.02	16.14	0.26	0.47
1.038	0.02	50.23	0.80	0.47
3.114	0.49	54.60	26.75	1.34
3.114	0.49	63.80	31.26	1.34
3.114	0.49	66.26	32.47	1.34
3.114	0.49	69.94	34.27	1.34
3.114	0.49	61.52	30.15	1.34
3.114	0.49	65.13	31.91	1.34
3.114	0.49	61.10	29.94	1.34
3.114	0.49	58.74	28.78	1.34
3.114	0.49	68.17	33.40	1.34
3.114	0.49	67.70	33.17	1.34
3.114	0.49	68.25	33.44	1.34
3.114	0.49	72.08	35.32	1.34

**Legend:** Dose (nmol/site); x_i_: Log of dose (nmol/site); y_i_: (% of MEP); x_i_y_i_: product x_i_.y_i_; (x_i_−$\overset{®}{X}$)^2^: (x_i_ –x_i_ averaged)^2^.

- Linear regression summary:

∑x= -54.8; ∑y= 3260; N= 82; $\overset{®}{X}$= 0.66822; $\overset{®}{Y}$= 40; ∑x^2^ = 87.4664;

∑x.y= -1089.7; N.x.y = -2178.55; $\overset{®}{X}$^2^= 0.446517

b= $\frac{\sum \mathrm{xiyi}-N\overset{®}{\mathrm{xy}}}{\Sigma {\mathrm{xi}}^{2}-N{\overset{®}{x}}^{2}}$;

*a* = $\overset{®}{y}$ - b$\overset{®}{x}$. thus *b* = 21.4 (slope) and *a* = 54.1 *(intersept)*

- Slope variance = $V(b)=\frac{s^{2}}{Ƹ\left(\mathrm{xi}-X\right)2}$. wherein s^2^ = *Q/(N-2*) and *Q* = $\sum_{i=1}^{N}(yi–a–\mathrm{bx}i)$

V(b) = 4.9

- Assessment if slope differs from 0:

t_*slope*_ = b/{V(b)}^1/2^; t_*slope*_ = 9.64 which exceeds t-table, thus slope differs from zero

t_*table*_ (N-2 degrees of freedom; P=0.05; from t-distribution) = 1.96

- DE_20_ calculation: DE20 = (20 – a)/b (in log scale); DE20 = −1.59 or 0.025 nmol/site

- Variance of DE_20_ (V_DE20_); = s^2^ / b^2^ [ 1/N + (x^1^−x¨)^2^/ S_xx_]; V_DE20_ = 0.016 (in log scale)

### 1.3.2 Phα1β (i.t.) combined with SB366791 (i.p.)

 See Table 7.

**Table 7:** Dose-effect data Phα1β (i.t.) and SB366791 (i.p.) on capsaicin-induced nociception.

Dose	x_i_	y_i_	x_i_y_i_	(x_i_−X̅)2
0.022	-1.66	2.00	-3.07	1.50
0.022	-1.66	-33.77	56.06	1.50
0.022	-1.66	32.95	-54.70	1.50
0.022	-1.66	-34.79	57.76	1.50
0.022	-1.66	29.24	-48.54	1.50
0.022	-1.66	32.19	-53.43	1.50
0.022	-1.66	49.14	-81.57	1.50
0.022	-1.66	5.42	-9.00	1.50
0.022	-1.66	23.73	-39.39	1.50
0.022	-1.66	37.13	-61.64	1.50
0.022	-1.66	52.16	-86.59	1.50
0.022	-1.66	34.40	-57.10	1.50
0.066	-1.18	15.30	-18.06	0.56
0.066	-1.18	51.72	-61.02	0.56
0.066	-1.18	50.92	-60.09	0.56
0.066	-1.18	25.35	-29.91	0.56
0.066	-1.18	-29.95	35.35	0.56
0.066	-1.18	34.40	-40.59	0.56
0.066	-1.18	39.56	-46.68	0.56
0.066	-1.18	49.14	-57.99	0.56
0.066	-1.18	24.75	-29.20	0.56
0.066	-1.18	32.88	-38.80	0.56
0.066	-1.18	34.40	-40.59	0.56
0.066	-1.18	26.88	-31.72	0.56
0.200	-0.69	27.18	-18.75	0.07
0.200	-0.69	5.01	-3.46	0.07
0.200	-0.69	54.88	-37.87	0.07
0.200	-0.69	14.98	-10.33	0.07
0.200	-0.69	-2.30	1.59	0.07
0.200	-0.69	50.23	-34.66	0.07
0.200	-0.69	52.09	-35.94	0.07
0.200	-0.69	46.93	-32.38	0.07
0.200	-0.69	68.30	-47.13	0.07
0.200	-0.69	31.86	-21.99	0.07
0.200	-0.69	40.00	-27.60	0.07
0.200	-0.69	59.32	-40.93	0.07
0.200	-0.69	35.08	-24.21	0.07
0.200	-0.69	34.00	-23.73	0.07
0.200	-0.69	55.58	-38.35	0.07
0.600	-0.22	62.01	-13.64	0.05
0.600	-0.22	37.47	-8.24	0.05
0.600	-0.22	35.88	-7.89	0.05
0.600	-0.22	70.28	-15.46	0.05
0.600	-0.22	54.38	-11.96	0.05
0.600	-0.22	53.69	-11.81	0.05
0.600	-0.22	54.30	-11.95	0.05
0.600	-0.22	53.56	-11.78	0.05
0.600	-0.22	65.36	-14.38	0.05
0.600	-0.22	41.02	-9.02	0.05
0.600	-0.22	36.95	-8.13	0.05
0.600	-0.22	59.32	-13.05	0.05
0.600	-0.22	52.16	-11.48	0.05
0.600	-0.22	41.23	-9.07	0.05
0.600	-0.22	56.95	-12.53	0.05
1.800	0.25	81.00	20.25	0.47
1.800	0.25	71.50	17.88	0.47
1.800	0.25	52.51	13.13	0.47
1.800	0.25	71.66	17.91	0.47
1.800	0.25	57.14	14.29	0.47
1.800	0.25	59.22	14.80	0.47
1.800	0.25	74.20	18.55	0.47
1.800	0.25	54.30	13.57	0.47
1.800	0.25	67.57	16.89	0.47
1.800	0.25	52.20	13.05	0.47
1.800	0.25	43.05	10.76	0.47
1.800	0.25	63.39	15.85	0.47
1.800	0.25	44.65	11.16	0.47
1.800	0.25	48.75	12.19	0.47
1.800	0.25	63.78	15.95	0.47
5.400	0.73	77.88	56.85	1.36
5.400	0.73	57.83	42.22	1.36
5.400	0.73	68.89	50.29	1.36
5.400	0.73	76.41	55.78	1.36
5.400	0.73	56.51	41.25	1.36
5.400	0.73	69.78	50.94	1.36
5.400	0.73	65.42	47.76	1.36
5.400	0.73	52.20	38.11	1.36
5.400	0.73	67.46	49.24	1.36
5.400	0.73	57.63	42.07	1.36
5.400	0.73	61.05	44.56	1.36
5.400	0.73	65.15	47.56	1.36

Legend: Dose (nmol/site); x_i_: Log of dose (nmol/site); y_i_: (% of MEP); x_i_y_i_: product x_i_.y_i_; (x_i_−$\overset{®}{X}$)^2^: (x_i_ –x_i_ averaged)^2^.

- Linear regression summary:

∑x= -35.22; ∑y= 3614; N=81; $\overset{®}{X}$= -0.43481; $\overset{®}{Y}$= 45; ∑x^2^ = 64.9758;

∑x.y= -573.761; N.x.y = -1571.51; $\overset{®}{X}$^2^= 0.1890

b= $\frac{\sum \mathrm{xiyi}-N\overset{®}{\mathrm{xy}}}{\Sigma {\mathrm{xi}}^{2}-N{\overset{®}{x}}^{2}}$;

*a* = $\overset{®}{y}$ - b$\overset{®}{x}$. thus *b* = 20.1 (slope) and *a* = 53.4 *(intersept)*

- Slope variance = $V(b)=\frac{s^{2}}{Ƹ\left(\mathrm{xi}-X\right)2}$. wherein s^2^ = *Q/(N-2*) and *Q* = $\sum_{i=1}^{N}(yi–a–\mathrm{bx}i)$

V(b) = 5.1

- Assessment if slope differs from 0:

t_*slope*_ = b/{V(b)}^1/2^; t_*slope*_ = 7.99 which exceeds t-tabulated. Thus, slope differs from zero

t_*table*_ (N-2 degrees of freedom; P=0.05; from t-distribution) = 1.96

- DE_20_ calculation: DE20 = (20 – a)/b (in log scale); DE20 = -1.66 or 0.022 nmol/site

- Variance of DE_20_ (V_DE20_); = s^2^ / b^2^ [ 1/N + (x^1^−x¨)^2^/ S_xx_]; V_DE20_ = 0.033 (in log scale)

## 1.4 Statistical comparisons between experimentally obtained DE_20_ (Z_mix_) and Theoretical additive DE_20_ (Z_add_)

– ***Calculation of the Z***_***add***_.
– Z_add_ = f.A + (1-f). B wherein:

f = the proportion factor (obtained from topic B);
A = DE_20_ of SB366791;
B = DE_20_ of Phα1β.

– Variance of Z_add_ = f^2^. V(A) + (1-f)^2^. V(B). wherein

f = proportion factor;
V(A) and V(B) = Variances of the DE_20_ of SB366791 and Phα1β, respectively.

– **Comparison test**
– t_*critical*_ = (x-y)/[(SEx)^2^+(SEy)^2^]^1/2^; wherein:

x = Log of Z_add_
y = Log of Z_mix_
(SEx)^2^ = V(Z_add_)
(SEy)^2^= V(Z_mix_)

– T_*tabulated*_ = [t_add_(SEx)_2_+t_mix_(SEy)^2^]/[(SEx)^2^+(SEy)^2^]; where in:

t_add_ = tabular value of t based on N´-2 degrees of freedom for 95% of significance
t_mix_ = tabular value of t based on N-2 degrees of freedom for 95% of significance
(SEy)^2^= Variance of Z_mix_

Interpretation: If t_*critical*_<T_*tabulated*_, then the difference is not significant, which means an additive effect. When t_*critical*_>T_*tabulated*_, Z_*mix*_ is significantly smaller (95% of confidence) than the Z_add_, which implies a synergistic effect of combined drugs (Table 8).

**Table 8:** Compilation of the comparison test.

Route of combined agents	Z_add_(nMol)	Z_mix_ (nMol)	t_*critical*_	T_*tabulated*_	Statistical significance
Intraplantar/Intraplanar	0.35	0.025	6.37	1.96	Yes
Intraplantar/Intrathecal	0.34	0.022	5.44	1.96	Yes

## 2 Experimental design, materials and methods

### 2.1 Capsaicin-induced nociceptive responses

This test was conducted essentially as described previously [1]. Briefly, individual animals were placed in transparent acrylic square boxes (20 cm per side) immediately after the capsaicin injection (1 nmol/paw i.p.), and nociceptive behaviours were recorded continuously for 300 s. Behaviours were quantified by recording the time spent licking, biting or flinching (nociceptive time) the paw injected with capsaicin. Phα1β (i.t. or i.p), SB366791 (i.p.), combined Phα1β + SB366791 or their respective vehicles were injected 10 min before the capsaicin injection. The initial dose ranges of Phα1β and SB366791 were selected based on previous data [1]. Antinociceptive effects were measured as a percentage of the maximum possible (% MPE) effect according to the formula: % MPE = 100*(A – B / A), where A is the averaged nociceptive time of the vehicle group and B is the nociceptive time of each animal in the treated group (drugs alone or in combination) [2].

### 2.2 Experimental design and statistical isobolographic analysis

Experimental design and statistical analyses were conducted essentially as previously described [3], [4], [5]. Briefly, dose-response curves were first obtained for Phα1β and SB366791 administered alone. Line equations, slope values and respective variances were obtained using linear regression [3]. DE_20_ values and 95% confidence limits were calculated using probit analysis. DE_20_ values (doses that exhibit 20% MPE) were used to assess whether the dose-effect of these drugs alone exhibited a constant potency ratio, which is necessary to perform fixed dose-pair combination of drugs [4].

Drug doses in association studies were determined as a proportion of their DE_20_ values. This proportion was constant and estimated based on a factor derived from the individual variances of the DE_20_ values. This fixed proportion of agents was necessary to assess whether the combination displayed enhanced potency indicative of synergism. Dose-response curves of associated drugs were constructed to obtain the doses that achieved the same effect level (20% MPE) compared to drugs given alone. This experimentally obtained DE_20_ (here called Z_mix_) was compared to theoretically calculated DE_20_ value for additive interactions (Z_add_). The criterion for establishing a statistical significance was P < 0.05. Graphical assessments of synergy are also presented using isobolographic analyses. Measurement of the interaction index (α) was obtained by dividing experimentally obtained DE_20_ of the drug pair by the theoretical additive DE_20_ of the drug pair. The α interaction index provides a measure of the degree of synergism.
