# Supplementary material for: Data and calculus on isobolographic analysis to determine the antinociceptive interaction between calcium channel blocker and a TRPV1 blocker in acute pain model in mice
Source: Data Brief. 2017 Jul 27;14:440–52. doi: 10.1016/j.dib.2017.07.059 (PMC5552379; doi:10.1016/j.dib.2017.07.059)
Supplement: Supplementary file 1 — Supplementary material [file mmc1.pdf]

CONFLICT OF INTEREST FORM

Article title: "Data and calculus on isobolographic analysis to determine the antinociceptive interaction between calcium channel blocker and a TRPV1 blocker in acute pain model in mice"

Manuscript number: **DIB-D-17-00543R1**

Author names: Juliana F. Silva, Manuella R. Palhares, Duana C. Santos, Cláudio A. Silva-Junior, Juliano Ferreira, Marcus V. Gomez, Célio J. Castro Junior

The authors declare that they have no conflicts of interest with respect to this report.

This work was supported by Fapemig CBB-RED-00006-14, CNPq Universal 456048/2014, FAPEMIG Universal APQ-01553-14, Capes Toxinology 1444/2011 and Capes Decit 2865/10.

Print name:

CÉLIO JOSÉ DE CASTRO JUNIOR

Signature:

Célio José de Castro Junior
____
